# Identifying Symptom Dynamics and Profiles of Subgroups at Risk for Major Depression and Suicidal Ideation Among Korean Adults: 2-Week Ecological Momentary Assessment Study

**DOI:** 10.2196/80332

**Published:** 2026-07-31

**Authors:** Kyungmi Chung, Jin Young Park

**Affiliations:** 1 Department of Psychiatry and Institute of Behavioral Science in Medicine, Yonsei University College of Medicine, Seoul, Republic of Korea; 2 Department of Psychiatry, Yongin Severance Hospital, Yonsei University College of Medicine, 363 Dongbaekjukjeon-daero, Giheung-gu, Yongin, 16995, Republic of Korea, 82 31-5189-8148; 3 Center for Digital Health, Yongin Severance Hospital, Yonsei University College of Medicine, Yongin, Republic of Korea

**Keywords:** depression, suicidal ideation, recall bias, mobile app, ecological momentary assessment, EMA, active monitoring, variability, cluster analysis

## Abstract

**Background:**

Traditional clinical assessments in psychiatric research or clinical practice rely on global retrospective self-report depression measures, which do not adequately capture intra- and interindividual variability in depressive symptoms over time and across contexts.

**Objective:**

This study aimed to (1) assess the sensitivity of mobile ecological momentary assessment (EMA) for monitoring depressive symptoms compared with traditional depression scales, (2) investigate changes in depressive symptoms and recall consistency observed between the first week (FW) and the second week (SW), and (3) identify subgroups at higher risk for depression and suicidal ideation, and characterize their sociodemographic, psychological, and psychiatric profiles.

**Methods:**

Participants’ self-reports were collected once daily for 14 consecutive days via a mobile app–based EMA to monitor the presence of 20 depressive symptoms based on a 24-hour recall period, thereby capturing naturalistic severity and variability. Baseline questionnaires measured sociodemographic characteristics, digital sensitivity, and personality traits. On day 14, postquestionnaires were administered to assess their clinical symptoms. In addition, modified depression scales were administered on day 7 for 1-week recall, and day 14 for both 1-week and 2-week recalls. For cluster analysis, 3 active EMA-derived features were included: mean symptom severity, within-person symptom variability, and frequency of suicidal ideation.

**Results:**

Generalized Linear Mixed Model analysis (model 1) revealed that traditional 2-week recall explained only 35.5% of the variance in daily symptom presence (odds ratio 0.666, 95% CI 0.659‐0.674; *P*<.001). Additional Generalized Linear Mixed Model analysis (model 2) identified a robust interaction, indicating that the consistency between weekly retrospective recall and daily EMA differed significantly between FW and SW (*F*_1, 81876_=124.550; *P*<.001). While overall symptom severity scores significantly decreased from FW to SW across both assessment methods (Cohen *d*=0.10‐0.34; *P*<.001), the estimated mean of the probability of symptom reporting showed a contrasting upward trend from 0.853 to 0.908. In cluster analysis, 695 participants (244 males and 451 females; aged 19‐73 years, mean 36.14, SD 10.71 years) who completed at least 7 EMA sessions over a 2-week period were classified into three distinct clusters: (1) no or low risk (n=445, 64.0%), (2) moderate risk (n=223, 32.1%), and (3) high risk (n=27, 3.9%). While depressive symptom severity, frequency of suicidal ideation, and psychiatric profiles progressively increased across clusters (cluster 1<2<3), the highest symptom variability was observed in cluster 2 (cluster 1<3<2).

**Conclusions:**

This study demonstrated that mobile EMA effectively reduced recall bias in assessing depressive symptoms and revealed symptom dynamics. Using a minimal set of active EMA-derived features, we differentiated 3 distinct risk clusters; within-person symptom variability emerged as a clinically significant indicator not captured by traditional 2-week recall–based assessments. Despite the brief 2-week assessment period, these findings suggest that mobile EMA may serve as a robust real-world data collection tool.

## Introduction

### Background

As one of the key symptoms of major depressive disorder (MDD), suicidal ideation is common among patients with MDD. The overall prevalence of suicidal ideation is estimated at 37.7% among patients with MDD [1]. While sustained depressed mood is not associated with lifetime suicidal ideation and suicide attempts, MDD can increase suicide risk regardless of the presence of sustained depressed mood [2]. Therefore, it is crucial to track the changes across all 9 symptoms of major depressive episode (MDE) meeting the *DSM-5* (*Diagnostic and Statistical Manual of Mental Disorders*, Fifth Edition) diagnostic criteria for MDD [3] rather than focusing solely on those in mood. Furthermore, depressive symptoms are the only risk factor that can differentiate the high-risk group with suicidal ideation and suicide attempt history from the nonsuicidal group [4]. The trajectory of depression symptoms with the highest mean scores and variability over time can be identified as a predictor of suicide attempts, particularly in young adults [5]. Since suicide is conceptualized as a process or a continuum of suicidal ideation or behaviors developing from mild to severe levels of suicidality among patients with MDD [6], monitoring and treating the day-to-day variations in depression symptoms and self-harm or suicidal ideation may lower the risk of suicidal behaviors. Among patients with MDD, the decline in suicidal ideation is preceded by the alleviation of depressive symptoms [7]. As suicidal ideation in remitted patients with MDD predicts the recurrence of depression, consistently measuring suicidal ideation among individuals with MDD, as well as among those with minimal suicidal ideation at any presentation, can also prevent the worsening of suicidal symptoms and recurrence of depression [8]. In this respect, the severity and variability of depressive symptoms should be measured alongside suicidal thoughts in daily life.

Unlike many previous studies that considered affective dynamics (ie, variability, inertia, and instability) as risk factors for depression [9-12], this study focuses more on the feasibility of identifying digital phenotypes that distinguish homogeneous and distinct subgroups at risk for major depression and suicidal ideation, as well as on developing an evidence-based measurement tool to minimize recall bias and facilitate enhanced recall of current MDE symptoms in everyday life [13,14]. According to the self-schema model of depression based on the concept of organizational breakdown and subsequent restoration to effective processing of schema-compatible information [15,16], nondepressives and clinical depressives with well-organized, efficient cognitive schemas showed higher self-referent yes recall for nondepressed content and depressed content, respectively, when compared with mild depressives in a state of uncertainty and disorganization concerning one’s self-schema. In line with this model, it can be postulated that mild depressives appear to contain both positive and negative personal information in their self-schema because they begin to view themselves with depressed (or negative) content although positive information has not yet been completely displaced. In this context, individuals with mild to moderate depression may show greater fluctuations in symptoms due to mixed self-schema representations, making variability a potential marker for identifying subgroups and their profiles. To explore different profiles in the dynamics of depressive symptoms, there is a need to address the limitations of standard depression scales, which rely on summated total scores based not only on classical test theory [17] but also on retrospective recall of symptom frequency over the preceding 2 weeks.

From the findings of previous studies, the issue of recall bias has been highlighted in ratings from traditional depression scales. More individuals reported severe depressive symptoms and higher suicidal ideation via ecological momentary assessment (EMA) than via retrospective self-report measures [18-20]. Moreover, more than half of the participants who reported suicidal ideation via EMA denied even past-week ideation on the scale for suicidal ideation, and the participants reporting suicidal ideation via EMA but not on the scale were just as likely to have a history of suicidal behavior as those reporting suicidal ideation in both formats [21]. In other words, EMA can capture instances of suicidal thoughts that go undetected via retrospective reports, thereby helping to identify at-risk subgroups that would otherwise be missed. Previous studies primarily demonstrated recall bias by comparing the total scores of depressive symptoms calculated from EMA data and those calculated from retrospective methods such as well-established interviewer- or self-administered measures. However, relatively little is known about how the frequency of depressive symptoms collected via EMA differs from that reported through traditional retrospective measures. To tackle these issues, we conducted a preliminary study, developing a self-administered 24-hour recall–based EMA tool to prospectively assess daily MDE symptoms over a 2-week period [13,14]. Specifically, this app uses the mobile version of the standardized Korean Version of the Center for Epidemiologic Studies Depression Scale–Revised (K-CESD-R), in which respondents are asked to report the presence of depressive symptoms within the past 24 hours by answering “yes” or “no” once daily at a self-selected notification time over a 2-week period. Taken together, using smartphone–based EMA technique (hereinafter, mobile EMA or active EMA) may allow for more accurate measurements of within- and between-participants variations in daily depressive symptoms, including self-harm and suicidal ideation, by reducing the recall period for symptom assessment.

Mobile EMA apps can enhance measurement precision and motivate repeated self-observation, thus increasing emotional reactivity and facilitating behavioral change, potentially reducing depressive symptoms [22,23]. Such symptom improvements may occur even without a formal intervention due to assessment reactivity, defined as symptom changes induced by repeated EMA assessments [24]. However, concerns remain that frequent self-monitoring might exacerbate negative affect and depressive symptoms [25,26]. Despite these concerns, previous evidence indicates that digital self-monitoring typically leads to enhanced emotional awareness, symptom reduction, and increased empowerment without triggering negative reactivity [27-29]. Although many studies have demonstrated the feasibility of using mobile EMA to capture moment-to-moment affective dynamics related to depression, relatively few studies have prospectively monitored MDEs using EMA [11,18]. Moreover, limited research exists on whether repeated self-monitoring itself directly contributes to measurable improvements in depressive symptoms.

### Objectives

The objectives of this study are (1) to assess the sensitivity of mobile EMA for monitoring depressive symptoms in daily life compared with traditional depression scales, (2) to investigate changes in depressive symptoms and recall consistency observed between the first week (FW) and the second week (SW), and (3) to identify homogeneous groups of community-dwelling adults at higher risk for depression and suicidal ideation and characterize their sociodemographic, psychological, and psychiatric profiles.

## Methods

### Study Design

In this study, we used a 2-week observational study design using mobile EMA and traditional retrospective scales. To address objectives 1 and 2, we administered a 24-hour recall–based mobile EMA once daily for 14 days and a modified version of the K-CESD-R in a 1-week recall format on day 7 and day 14, as well as in a 2-week recall format on day 14, reflecting the K-CESD-R reporting frame (“in the past week or so,” including an option reflecting “nearly every day for 2 weeks”). To further address objective 3, we restricted our cluster analysis to active EMA data-driven features (symptom severity, symptom variability, and frequency of suicidal ideation) that could be extracted even if participants did not respond every day during the 2-week study period, given that greater depressive symptom severity might be associated with lower adherence to digital intervention for depression [30].

### Participant Recruitment

As a fully decentralized clinical trial, this study was conducted in collaboration with dataSpring Korea, Inc., over an approximately 1-month period from August 16, 2023, to September 18, 2023. A total of 1049 community-dwelling adults were recruited through PanelNow, a proprietary online research panel service operated by dataSpring Korea.

#### Eligibility and Dropout Criteria

In accordance with the institutional review board (IRB)–approved protocol, potential participants were assessed based on the following inclusion and exclusion criteria. This study required participants to meet all the following inclusion criteria: a person (1) who checks “Agree” on the e-consent form presented at the beginning of the online survey, (2) who is aged 19 years and older, (3) who owns a smartphone registered in their name and is able to install the mobile app used in this study, and (4) whose smartphone operating system meets the minimum requirements to install the app. However, even if all the inclusion criteria are met, people for whom (1) the mobile app is not properly installed and does not work on their smartphones or (2) the app does not run due to unknown errors that occur after completing its installation are not enrolled according to the exclusion criteria.

Participants were considered to have dropped out or were discontinued from the study if they met any of the following criteria: (1) failure to complete all mandatory prequestionnaires within the designated period; (2) never completing a single session after registering for the mobile EMA app; (3) inadequate adherence to the research protocol, defined as completing fewer than 7 out of the 14 scheduled EMA sessions or failing to complete all 3 traditional assessments; (4) providing responses judged to be insincere or unreliable; or (5) expressing a voluntary withdrawal of consent to participate in the study at any point. For participants who withdrew their consent or failed to meet the adherence criteria, all response data were immediately deleted from the server and not used for any analyses, with only basic app account logs and study participation logs retained for the legally mandated period.

### Ethical Considerations

To conduct this study, the study protocol was approved by the IRB of Yongin Severance Hospital, Yonsei University College of Medicine (IRB number 9-2023-0122). All participants provided electronic informed consent (e-consent) prior to their involvement in the study. The study information and consent form were presented online via the PanelNow platform and mobile app. Potential participants were required to sign in to their registered accounts to access the study link, where they read a comprehensive description detailing the research purpose, procedures, potential benefits and risks, and compensation. Consent was obtained voluntarily when participants checked an “Agree” radio button, which allowed them to proceed to the screening and baseline questionnaires. This e-consent explicitly covered the collection and processing of personal and sensitive data, including sociodemographic characteristics, clinical symptoms, and digital phenotypes. Participants were clearly informed of their right to withdraw from the study at any time for any reason without any disadvantage or penalty. They were also provided with instructions on how to request the permanent deletion of their previously collected data by contacting the PanelNow customer center should they choose to opt out. To protect participant privacy and confidentiality, all datasets were deidentified by the online panel service provider (dataSpring Korea) before being transferred to the research team; directly identifiable information (birth date and mobile phone number) was removed, providing only the minimum necessary variables (sex and age) for statistical analysis. The deidentified data are stored on a secure, password-protected computer and server whose access is restricted to authorized individuals directly involved in the study. To ensure multilayered security, separate passwords were set for reading and writing files, and these passwords were communicated through independent channels and updated periodically. Furthermore, the mobile EMA app used a secure protocol where user identities were converted into nonidentifiable IDs; it was technically impossible to decode these IDs back into identifiable personal information. Compensation was provided via “PanelNow” points (1 point=1 KRW [1 KRW=US $0.00075 as of September 19, 2023]), a redeemable reward unit used within the service platform. Eligible participants who completed all tasks received 6000 points; those with 100% adherence to the mobile EMAs received an additional 1000 points. The total possible compensation of 7000 points was equivalent to 7000 KRW (US $5.27).

### Traditional Retrospective Measure

#### Modified K-CESD-R

To determine whether recall bias affects the retrospective report of depressive symptoms on the standardized CESD-R assessment [31], we modified the K-CESD-R scale [32] to additionally require participants to specify the number of days they experienced the symptoms and to input numerical values for their responses into the modified format. Specifically, a 2-week recall was conducted on day 14 to evaluate the sensitivity of mobile EMA compared with traditional retrospective recall. To investigate changes in depression symptoms and recall consistency, a 1-week recall was also administered on day 7 (for first week, FW) and day 14 (for second week, SW). Furthermore, the modified scale was designed to prevent insincere random responses. If inconsistencies were detected between the responses on the original scale and the directly entered values, the survey system displayed a warning pop-up to reduce careless entries.

The K-CESD-R is a 20-item self-report measure of depressive symptoms in 9 different domains: sadness/dysphoria (questions 2, 4, and 6), loss of interest/anhedonia (questions 8 and 10), appetite (questions 1 and 18), sleep (questions 5, 11, and 19), thinking/concentration (questions 3 and 20), guilt/worthlessness (questions 9 and 17), tiredness/fatigue (questions 7 and 16), movement/agitation (questions 12 and 13), and suicidal ideation (questions 14 and 15). Participants are asked to rate each item on a 5-point scale to indicate how they felt or behaved during the last week or so: 0=not at all or less than 1 day in the last week, 1=1‐2 days in the last week, 2=3‐4 days in the last week, 3=5‐7 days in the last week, and 4=nearly every day for 2 weeks. All responses are summed for a total score ranging from 0 to 80, with scores of 13 or higher indicating the need for individuals to seek further clinical diagnostic interview. In this study, the total scores from the modified K-CESD-R, administered separately on day 7 and day 14 to assess the preceding 1-week recall periods, ranged from 0 to 60 rather than the original 0 to 80.

### EMA-Based Retrospective Measure

#### Mobile App–Based Depression Assessment

**Figure 1.**
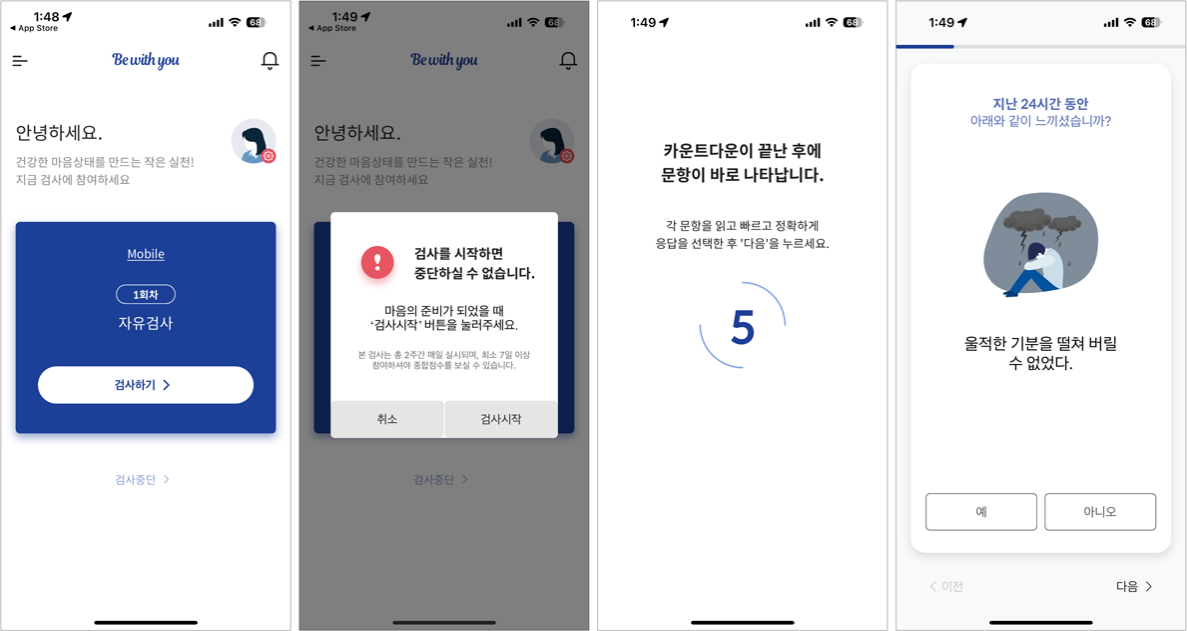
Screenshots of the 24-hour recall-based depression assessment procedure in the BeWithYou app.

**Figure 2.**
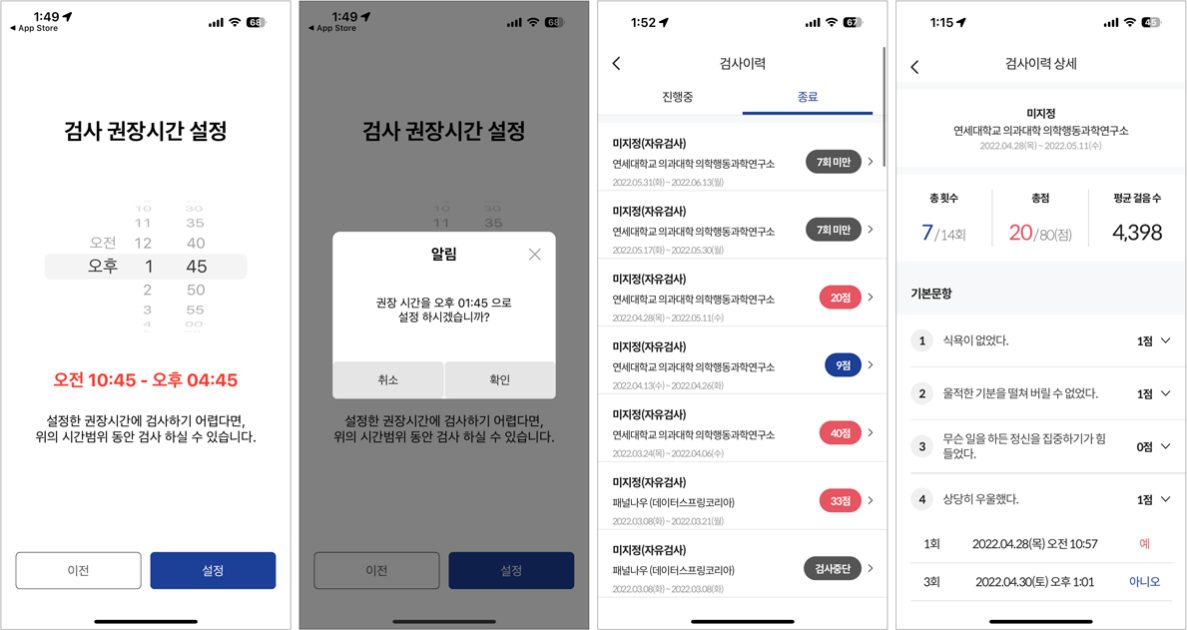
Screenshots of the preferred assessment time reminder feature and assessment history menu in the BeWithYou app.

The “BeWithYou” app, an upgraded version of the K-CESD-R mobile app developed in our previous study [13], is designed as a 24-hour recall–based prospective depression assessment tool. A single test set consists of 14 sessions over a 2-week period and can be repeatedly administered to monitor the improvement and deterioration of depressive symptoms in individuals with or with no previous diagnosis of major depression. In the test set, 20 items are presented in a randomized order in each session, and participants respond either “yes (1=presence)” or “no (0=absence)” to each item (eg, “In the past 24 hours, I felt depressed”) (Figure 1). At the start of the first session, the app allows participants to set a daily assessment reminder, ensuring that they can report their symptoms regularly during this study period (Figure 2). Regarding the clinically validated scoring algorithm embedded in the app [13], the total score is automatically calculated only if participants complete at least 7 sessions. If they complete fewer than 7 sessions, only the total number of tests taken over the 2 weeks and the number of days each symptom is reported can be viewed in the main menu of the assessment history (Figure 2).

### Psychological and Psychiatric Measures

#### Digital Sensitivity

The Yongin Severance Digital Sensitivity Scale, developed by Park et al [33], is a self-report instrument designed to measure individual differences in digital sensitivity. The scale comprises 6 subfactors across 2 domains: digital literacy (14 items) and digital efficacy (6 items). The digital literacy domain includes 4 subfactors: digital application (4 items), digital communication (3 items), critical thinking (4 items), and digital ethics (3 items). The digital efficacy domain consists of digital confidence (3 items) and digital anxiety (3 items). All items are rated on a 7-point Likert scale ranging from 1 (strongly disagree) to 7 (strongly agree), yielding a total score ranging from 20 to 140. Items in the digital anxiety subscale are reverse-scored before computing the total score. Higher total scores indicate higher levels of digital sensitivity.

#### Personality Traits

To quickly and easily measure the Big Five personality traits, we used a Korean version of the brief Big Five Inventory-15 [34], which was adapted and shortened to 15 items from the 44-item Big Five Inventory [35]. The Korean version of the brief Big Five Inventory-15 comprises 15 items across 5-factor personality domains: (1) neuroticism (3 items), (2) extraversion (3 items), (3) openness (3 items), (4) conscientiousness (3 items), and (5) agreeableness (3 items). Each item is rated on a 5-point scale (1=strongly disagree to 5=strongly agree). The responses to the 3 items within each domain were averaged for the data analysis.

#### Depression

As a well-validated multipurpose instrument for screening and diagnosing major depression, the Patient Health Questionnaire–9 (PHQ-9 [36]) is adopted to measure the presence and severity of 9 MDE symptoms over the past 2 weeks. On the last day of the 2-week study period, we administered the Korean version of PHQ-9 scale [37] to collect these data, aligning them with the data measured through the modified K-CESD-R scale and the BeWithYou app. As each of the 9 items can be scored from 0 (not at all) to 3 (nearly every day), the total score of the PHQ-9 ranges from 0 to 27 (≤4: no depression, 5‐9: mild depression, 10‐19: moderate depression, and ≥20: severe depression), with scores of 10 or greater indicating clinically significant depression.

#### State-Trait Anxiety

As a measure of the severity of anxiety, the Spielberger State-Trait Anxiety Inventory (STAI-form X [38]) was adopted to separately measure 2 types of anxiety: state anxiety (ie, how you feel right now, ie, at this moment) and trait anxiety (ie, how you generally feel). In this study, we administered a revised version of the STAI [39], a 40-item self-report questionnaire with 2 subscales: the STAI-X-1 (state anxiety) and the STAI-X-2 (trait anxiety). Both subscales consist of 20 items each, rated on a 4-point scale with different anchors for the STAI-X-1 (1=not at all, 2=somewhat, 3=moderately so, and 4=very much so) and the STAI-X-2 (1=almost never, 2=sometimes, 3=often, and 4=almost always). All responses to positively stated items are reverse-coded to calculate each of the total scores for the STAI-X-1 (items 1, 2, 5, 8, 10, 11, 15, 16, 19, and 20) and the STAI-X-2 (items 1, 6, 7, 10, 13, 16, and 19); after that, the total scores of the STAI-X-1 and the STAI-X-2 are calculated by summing the responses to the 20 items for each subscale. The total scores range from 20 to 80, with higher scores indicating greater levels of anxiety.

#### Insomnia

As a brief self-report instrument measuring subjective insomnia complaints in the last 2 weeks, the Insomnia Severity Index (ISI [40]) consists of 7 items: (1) the severity of sleep-onset (1a: initial), sleep maintenance (1b: middle), and early morning awakening (1c: terminal) difficulties; (2) the dissatisfaction with current sleep pattern (dissatisfaction); (3) the interference with daily functioning (interference); (4) the noticeability of impairment in the life quality attributed to the sleep problem (noticeability); and (5) the degree of concerns or distress caused by the current sleep problem (distress). Each of the ISI items is rated on a 5-point scale with different anchors from 0 to 4, and scores are summed to yield a total score ranging from 0 to 28, with high scores indicating greater insomnia severity. The total scores are interpreted as follows: no clinically significant insomnia (0‐7), subthreshold insomnia (8-14), clinical insomnia (moderate, 15‐21), and clinical insomnia (severe, 22‐28). In this study, we administered the Korean version of the Insomnia Severity Index (ISI-K), standardized and validated by Cho et al [41]. A cutoff score of 15 on the ISI-K is used as the threshold for clinically significant insomnia.

#### Somatic Symptoms

In this study, somatic symptoms were assessed using the Korean version of the Patient Health Questionnaire–15 [42]. This instrument consists of 15 items that ask how often respondents have been bothered by various physical symptoms over the past 4 weeks, rated on a 3-point Likert scale ranging from 0 (not at all) to 2 (bothered a lot). Total scores range from 0 to 30, with higher scores indicating more severe somatic symptom burden. A cutoff score of 8 or above was used to indicate clinically relevant somatization.

### Sociodemographic Measures

Sociodemographic measures included sex, date of birth, educational attainment, marital status, number of other household members, and subjective socioeconomic status (SES), all of which were collected by self-report. Sex was assessed as a dichotomous variable (male or female). Using the date of informed consent as the reference date, age was automatically calculated from the participant’s self-reported date of birth. Educational attainment was measured as the highest level of education completed, with response options of no formal education, elementary school, middle school, high school, 2‐ to 3-year college, 4-year university or higher, master’s degree, doctoral degree, or other. Marital status was measured using 6 categories: single, cohabiting or common-law marriage, married, separated, divorced, or widowed. A current living arrangement variable was derived from the reported number of other household members and categorized into none (1-person household) through 5 or more people (6-or-more-person household). For analysis, this variable was recategorized as living alone (1-person household) or living with others (≥2-person household). SES was assessed with a single item asking participants to rate their overall socioeconomic position considering occupation, educational level, income, and assets on a 5-point scale ranging from very low to very high.

### Procedure

In this study, potential participants were recruited via PanelNow, a mobile panel platform directly operated by dataSpring Korea. Access to the study was restricted to registered app users, who received an in-app notification with an embedded link redirecting them to the e-consent form and online prequestionnaires. These prequestionnaires consisted of screening items to determine eligibility, followed by baseline questions regarding sociodemographic information, digital sensitivity, and personality traits. After providing e-consent, individuals who met all eligibility criteria and successfully completed the BeWithYou app installation and registration process were enrolled.

During the 2-week study period, enrolled participants followed a protocol consisting of four assessment components: (1) a first traditional assessment (1-week recall), (2) a second traditional assessment (both 1-week recall and 2-week recall), (3) a mobile app–based active EMA, and (4) final postquestionnaires. To ensure a rigorous evaluation of each component, specific protocols were established as follows. For traditional retrospective assessments, modified versions of K-CESD-R were administered on day 7 and day 14. Concurrently, the mobile EMA was conducted daily for 14 sessions. To be eligible for the final data analysis, participants were required to meet minimum adherence criteria, defined as completing at least 7 mobile EMA sessions and all 3 traditional assessments. On day 14, only those who fulfilled these requirements were provided with the link to the final postquestionnaires, which assessed clinical symptoms such as anxiety, depression, insomnia, and somatization. Except for the daily mobile EMA using the BeWithYou app, all questionnaires were administered online via URL links sent to the contact channels (including app push, email, or SMS text messaging) for which participants had maintained opt-in status for research participation notification.

### Statistical Analysis

All statistical analyses were performed using IBM SPSS Statistics (version 29.0.2.0 for Mac; IBM Corp), with 2-tailed *P* value of .05 considered statistically significant. Missing EMA sessions were retained as missing values so that all available repeated observations could be included in the analyses. To address objectives 1 and 2, we fitted 2 Generalized Linear Mixed Models (GLMMs), with a binomial distribution and logit link to predict the outcomes of daily EMA (“Symptom Presence”; 0=no and 1=yes). Retrospective K-CESD-R responses were first aggregated into 9 MDE symptom domains using the maximum number of days reported within each domain (eg, if “Sadness” items [3 items] were reported as 2, 0, and 5 days, the value “5” was used for that domain). In both models, “participant ID” was included as a random intercept to account for the nonindependence of repeated observations and control for stable between-person differences in reporting tendencies. Model 1 examined the association between daily EMA symptom presence (EMA sensitivity) and 2-week retrospective recall (systematic recall bias), with fixed effects of “Symptom Days_T2W” (0‐14) as a continuous covariate and “Day” (1-14) and “Domain” (1-9) as categorical factors. Model 2 further examined week-to-week differences in recall consistency using 1-week retrospective recall. Fixed effects included “Symptom Days_1W” (0‐7) as a continuous covariate and “Week” (FW vs SW), “Day” (1‐7, nested within week), and “Domain” as categorical factors. The “Week” × “Symptom Days_1W” interaction was also included to test whether recall consistency differed between weeks. Model fit was evaluated using the Akaike Information Criterion Corrected (AICC) and Bayesian Information Criterion (BIC). Variance explained was estimated using marginal (fixed effects only) and conditional (fixed and random effects) pseudo *R*^2^ values. The magnitude of between-person variability was assessed using the intraclass correlation coefficient (ICC). Finally, we conducted the paired samples *t* test to compare modified K-CESD-R total scores obtained on day 7 and day 14, representing the first and second 1-week recall periods, respectively. This comparison was intended to assess whether repeated EMA–based self-assessments in daily life were associated with positive changes in depression severity.

To address objective 3, K-means clustering was conducted using 3 *z*-standardized EMA-derived features: (1) mean number of daily depressive symptoms over the 2-week period (symptom severity: 0‐20 symptoms), (2) within-person SD of daily depressive symptom counts (symptom variability), and (3) mean number of days with suicidal ideation (items 14 and 15, frequency of suicidal ideation: 0‐14 days) (Table 1). We compared 3-, 4-, and 5-cluster solutions and selected the 3-cluster model based on silhouette coefficients and clinical interpretability. To validate the extracted clusters, 1-way ANOVA tests were performed using the cluster assignment as the independent variable and the means of the 3 EMA features as the dependent variables. Cluster differences in sociodemographic, psychological, and psychiatric profiles were evaluated using 1-way ANOVA, chi-square tests, and Bonferroni-adjusted post hoc comparisons (*P*<.05). Cramér *V* was used as the effect size for chi-square tests, with values of 0.10, 0.30, and 0.50 typically interpreted as indicating small, medium, and large effects, respectively [43].

**Table 1.** Algorithm used to calculate active EMA^a^-based features from participants’ responses to the K-CESD-R^b^ mobile scale.

Domain	2-week EMA protocol (day)														Total mean number of days with symptoms per domain over 2 weeks, day
	1	2	3	4	5	6	7	8	9	10	11	12	13	14	
Sadness (dysphoria)															
Item 2	0/1	0/1	0/1	0/1	0/1	0/1	0/1	0/1	0/1	0/1	0/1	0/1	0/1	0/1	0‐14
Item 4	0/1	0/1	0/1	0/1	0/1	0/1	0/1	0/1	0/1	0/1	0/1	0/1	0/1	0/1	
Item 6	0/1	0/1	0/1	0/1	0/1	0/1	0/1	0/1	0/1	0/1	0/1	0/1	0/1	0/1	
Loss of interest (anhedonia)															
Item 8	0/1	0/1	0/1	0/1	0/1	0/1	0/1	0/1	0/1	0/1	0/1	0/1	0/1	0/1	0‐14
Item 10	0/1	0/1	0/1	0/1	0/1	0/1	0/1	0/1	0/1	0/1	0/1	0/1	0/1	0/1	
Appetite															
Item 1	0/1	0/1	0/1	0/1	0/1	0/1	0/1	0/1	0/1	0/1	0/1	0/1	0/1	0/1	0‐14
Item 18	0/1	0/1	0/1	0/1	0/1	0/1	0/1	0/1	0/1	0/1	0/1	0/1	0/1	0/1	
Sleep															
Item 5	0/1	0/1	0/1	0/1	0/1	0/1	0/1	0/1	0/1	0/1	0/1	0/1	0/1	0/1	0‐14
Item 11	0/1	0/1	0/1	0/1	0/1	0/1	0/1	0/1	0/1	0/1	0/1	0/1	0/1	0/1	
Item 19	0/1	0/1	0/1	0/1	0/1	0/1	0/1	0/1	0/1	0/1	0/1	0/1	0/1	0/1	
Thinking/ concentration															
Item 3	0/1	0/1	0/1	0/1	0/1	0/1	0/1	0/1	0/1	0/1	0/1	0/1	0/1	0/1	0‐14
Item 20	0/1	0/1	0/1	0/1	0/1	0/1	0/1	0/1	0/1	0/1	0/1	0/1	0/1	0/1	
Guilt (worthlessness)															
Item 9	0/1	0/1	0/1	0/1	0/1	0/1	0/1	0/1	0/1	0/1	0/1	0/1	0/1	0/1	0‐14
Item 17	0/1	0/1	0/1	0/1	0/1	0/1	0/1	0/1	0/1	0/1	0/1	0/1	0/1	0/1	
Tiredness (fatigue)															
Item 7	0/1	0/1	0/1	0/1	0/1	0/1	0/1	0/1	0/1	0/1	0/1	0/1	0/1	0/1	0‐14
Item 16	0/1	0/1	0/1	0/1	0/1	0/1	0/1	0/1	0/1	0/1	0/1	0/1	0/1	0/1	
Movement (agitation)															
Item 12	0/1	0/1	0/1	0/1	0/1	0/1	0/1	0/1	0/1	0/1	0/1	0/1	0/1	0/1	0‐14
Item 13	0/1	0/1	0/1	0/1	0/1	0/1	0/1	0/1	0/1	0/1	0/1	0/1	0/1	0/1	
Suicidal ideation															
Item 14	0/1	0/1	0/1	0/1	0/1	0/1	0/1	0/1	0/1	0/1	0/1	0/1	0/1	0/1	0‐14
Item 15	0/1	0/1	0/1	0/1	0/1	0/1	0/1	0/1	0/1	0/1	0/1	0/1	0/1	0/1	
Total number of depressive symptoms per day (minimum-maximum), n	0‐20	0‐20	0‐20	0‐20	0‐20	0‐20	0‐20	0‐20	0‐20	0‐20	0‐20	0‐20	0‐20	0‐20	N/A^c^
Transformed score per item based on the BeWithYou scoring algorithm (minimum-maximum), point	0‐4	0‐4	0‐4	0‐4	0‐4	0‐4	0‐4	0‐4	0‐4	0‐4	0‐4	0‐4	0‐4	0‐4	N/A
Total score (minimum–maximum), point	0‐80														N/A

a EMA: ecological momentary assessment.

b K-CESD-R: Korean Version of the Center for Epidemiologic Studies Depression Scale–Revised.

c N/A: not applicable.

## Results

### Participant Flow and Sociodemographic Characteristics

**Figure 3.**
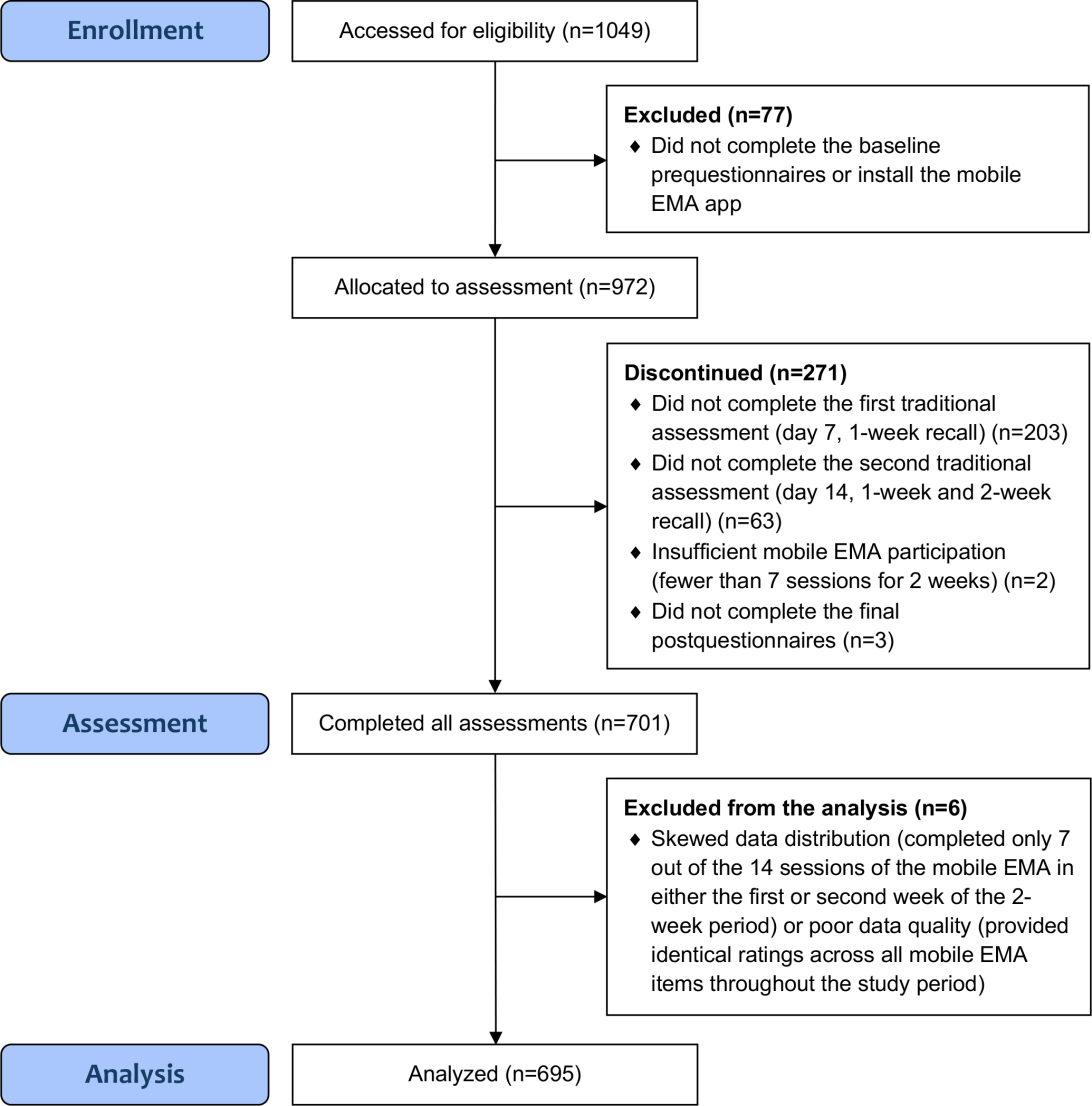
CONSORT (Consolidated Standards of Reporting Trials) flow diagram of this study. EMA: ecological momentary assessment.

The participant flow from initial recruitment to the final analysis is illustrated in Figure 3. Out of 1049 participants who were initially screened, 701 met all eligibility and adherence criteria and were included in the data analysis, while 348 were excluded based on predefined dropout criteria. Even if they met the minimum requirement of 7 sessions, we further excluded 6 participants who exhibited highly skewed or nonrepresentative response patterns that could undermine the clinical and statistical validity of the study.

Specifically, these exclusions included (1) participants whose responses were concentrated exclusively within a single week (either the FW or the SW), which not only precluded the assessment of the temporal continuity of depressive symptoms over the required 2-week period—a critical clinical criterion—but also rendered them ineligible for the planned comparison between the FW and the SW; and (2) participants who exhibited straight-lining EMA response patterns with zero variance, suggesting insincere participation. After these final exclusions, the sociodemographic and psychological characteristics of the final analytic sample (N=695) are summarized in Table 2.

**Table 2.** Sociodemographic and psychological characteristics of the total sample.

Characteristics	Total sample (N=695)
Sex, n (%)	
Male	244 (35.1)
Female	451 (64.9)
Age (years), mean (SD)	36.14 (10.71)
Age (years), range (minimum-maximum)	19‐73
Age group (years), n (%)	
19‐29	224 (32.2)
30‐39	215 (30.9)
40‐49	165 (23.7)
50‐59	75 (10.8)
60‐69	16 (2.3)
Educational attainment, n (%)	
High school	109 (15.7)
Associate degree (2‐3-year college)	118 (17.0)
Bachelor’s degree (4-year university or higher)	409 (58.8)
Master’s degree	50 (7.2)
Doctoral degree	9 (1.3)
Marital status, n (%)	
Single	354 (50.9)
Cohabiting/common-law marriage	16 (2.3)
Married	316 (45.5)
Previously married (separated/divorced/widowed)^a^	9 (1.2)
Current living arrangement, n (%)	
Living alone (1-person household)	109 (15.7)
Living with others (2 or more household members)	586 (84.3)
Subjective socioeconomic status, n (%)	
Lowest	21 (3.0)
Low	177 (25.5)
Middle	450 (64.7)
High	45 (6.5)
Highest	2 (0.3)
Personality trait, mean (SD)	
Openness to experience	3.16 (0.86)
Conscientiousness	3.81 (0.67)
Neuroticism	2.94 (0.92)
Extraversion	2.72 (0.85)
Agreeableness	3.46 (0.64)
Digital sensitivity, mean (SD)	90.12 (14.03)
Smartphone operating system, n (%)	
iOS	244 (35.1)
Android	451 (64.9)

a Previously married was defined as having been legally married at some point, including those currently separated, divorced, or widowed.

### Final Analytic Sample and Mobile EMA Outcome Events

Of the total 87,570 potential sessions, 81,900 (93.5%) valid EMA observations from 695 participants were included in the final analysis. This high inclusion rate, achieved by using all available data points through a GLMM, effectively mitigated potential selection bias and preserved statistical power despite occasional missing daily entries.

### Assessing Recall Bias in Traditional vs Mobile EMA–Based Depression Measures

The primary objective of model 1 was to quantify the systematic discrepancy and information loss inherent in traditional retrospective reports (2-week recall) compared with daily EMA records (24-hour recall). The final GLMM demonstrated robust statistical fit (AICC=473,906.603 and BIC=473,915.916). The overall classification accuracy for model 1 was 88.3%. Omnibus tests for fixed effects revealed that traditional retrospective reports (“Symptom Days_T2W”) were a statistically significant predictor of daily symptom presence (“Symptom Presence”; *F*_1, 81877_=4,826.322; *P*<.001). Significant main effects were also observed for “Domain” (*F*_8, 81877_=284.483; *P*<.001) and “Day” (*F*_13, 81877_=118.344; *P*<.001). Parameter estimates for “Symptom Days_T2W” yielded a significant negative slope (*Β*=−0.406, SE 0.006; *P*<.001), with an odds ratio of 0.666 (95% CI 0.659‐0.674). The marginal pseudo *R*^2^ value was 0.355, indicating that the traditional retrospective recall method explains only 35.5% of the variance in actual daily symptom presence. When accounting for random intercepts, the conditional pseudo *R*^2^ value increased to 0.504, with an adjusted ICC of 0.232. These results show that while interindividual heterogeneity accounts for 23.2% of the total variance, nearly 76.8% of the symptomatic variability is driven by intraindividual fluctuations, approximately half of which (49.6%) remains entirely unexplained by the 2-week recall method.

### Investigating Temporal Dynamics and Interaction Effects of Recall Periods

The second objective of model 2 was to investigate whether the consistency of recall remained stable or exhibited temporal fluctuations across the assessment period by comparing the FW and the SW. The final GLMM also demonstrated a robust statistical fit (AICC=469,917.005 and BIC=469,926.318) and achieved an overall classification accuracy of 88.5%. Omnibus tests for fixed effects revealed significant main effects for all predictors, including “Domain” (*F*_8, 81876_=276.180; *P*<.001), “Day” (*F*_12, 81876_=56.555; *P*<.001), and “Symptom Days_1W” (*F*_1, 81876_=7146.780; *P*<.001). Notably, a significant main effect of “Week” was also observed (*F*_1, 81876_=50.317; *P*<.001), with the estimated mean of “Symptom Presence” increasing from 0.853 (FW) to 0.908 (SW). Similar to model 1, parameter estimates for “Symptom Days_1W” yielded a significant negative slope (*Β*=−0.749, SE 0.011, *P*<.001; odds ratio 0.473, 95% CI 0.463‐0.483). The marginal pseudo *R*^2^ value of 0.335 indicates that the fixed effects, including the temporal interaction, explain only about one-third of the variance in daily symptom reporting. When accounting for random intercepts, the conditional pseudo *R*^2^ value increases to 0.475, indicating that more than 52% of the total variance in daily symptom presence remains entirely unexplained even when considering individual differences. The adjusted ICC of 0.211 further revealed that while interindividual heterogeneity accounts for 21.1% of the total variance, the vast majority of symptomatic variability, specifically nearly 79%, is attributable to within-person fluctuations. Most importantly, a robust interaction was found between “Week” and “Symptom Days_1W” (*F*_1, 81876_=124.550; *P*<.001). This significant interaction revealed that the consistency between weekly retrospective recall and daily EMA significantly differed between FW and SW.

### Evaluating Week-to-Week Changes in Depression Severity Across Recall Methods

To further elucidate the symptomatic trajectory observed in model 2, a supplementary analysis was conducted to compare the change in depression severity scores between FW and SW across 2 different assessment methods: a 1-week recall–based version (original) and a 24-hour recall–based version (mobile). Paired samples *t* tests revealed that the original scores significantly decreased from 12.46 (SD 13.54) in the FW to 11.17 (SD 13.76) in the SW (*t*_694_=5.41, Cohen *d*=0.10; *P*<.001). Additionally, a comparable reduction was observed in the mobile scores, which decreased from 14.29 (SD 13.71) to 11.11 (SD 14.03) (*t*_694_=13.04, Cohen *d*=0.34; *P*<.001). Notably, this overall reduction in symptomatic severity across both assessment methods stands in direct contrast to the upward trend in symptom reporting probability observed in model 2.

### Identifying and Profiling High-Risk Subgroups Using Active EMA Features

A K-means cluster analysis (*k*=3) was performed using active EMA features—symptom severity, symptom variability, and frequency of suicidal ideation—which were standardized using a *z*-score transformation. In the analysis, a total of 695 participants were identified as 3 distinct risk groups, and based on their symptom profiles, each cluster was subsequently labeled as no- or low-risk (cluster 1), moderate-risk (cluster 2), and high-risk (cluster 3) groups. Cluster 1 (n=445) was characterized by low symptom severity (*z*=−0.57; raw mean 1.07, SD 1.15), low symptom variability (*z*=−0.56; raw mean 0.95, SD 0.65), and minimal suicidal ideation (*z*=−0.30; raw mean 0.02, SD 0.12). Cluster 2 (n=223) had moderate symptom severity (*z*=0.79; raw mean 6.42, SD 3.20), the highest symptom variability (*z*=1.04; raw mean 3.07, SD 1.16), and occasional suicidal ideation (*z*=0.07; raw mean 0.62, SD 0.98). Cluster 3 (n=27) showed the highest symptom severity (*z*=2.80; raw mean 14.34, SD 2.13), moderate symptom variability (*z*=0.36; raw mean 2.17, SD 1.55), and frequent suicidal ideation (*z*=4.23; raw mean 7.37, SD 2.82). While both the severity of depressive symptoms and frequency of suicidal ideation, as well as all psychiatric characteristics, increased with the level of risk in the clusters (cluster 1<2<3), the highest variability of depressive symptoms was observed in cluster 2 (cluster 1<3<2).

To assess the robustness of the clustering outcomes, 1-way ANOVAs were conducted using the cluster assignments as the independent variable. Results confirmed significant between-group differences across all 3 EMA indices: *F*_2, 692_=892.24, partial *η*^2^=0.72, *P*<.001, for symptom severity; *F*_2, 692_=423.18, partial *η*^2^=0.55, *P*<.001, for symptom variability; and *F*_2, 692_=1117.36, partial *η*^2^=0.76, *P*<.001, for frequency of suicidal ideation. These findings support the internal validity of the 3-cluster classification.

#### Sociodemographic and Psychological Characteristics Across Clusters

As presented in Table 3, the sociodemographic and psychological characteristics are summarized for each of the 3 clusters identified in the cluster analysis: (1) cluster 1 (no or low risk: 445/695, 64.0%), (2) cluster 2 (moderate risk: 223/695, 32.1%), and (3) cluster 3 (high risk: 27/695, 3.9%).

**Table 3.** Sociodemographic and psychological characteristics of 3 clusters^a^.

Characteristics	Cluster 1 (n=445)	Cluster 2 (n=223)	Cluster 3 (n=27)	*P* value
Sex, n (**%**)				.20
Male	167 (37.5)	68 (30.5)	9 (33.3)	—^b^
Female	278 (62.5)	155 (69.5)	18 (66.7)	—
Age (years), mean (SD)	36.64 (10.89)	35.59 (10.42)	32.37 (9.25)	.09
Age (years), range (minimum–maximum)	19‐73	19‐69	19‐52	—
Age group (years), n (%)				.39
19‐29	141 (31.7)	72 (32.3)	11 (40.7)	—
30‐39	127 (28.5)	78 (35.0)	10 (37.0)	—
40‐49	114 (25.6)	48 (21.5)	3 (11.1)	—
50‐59	50 (11.2)	22 (9.9)	3 (11.1)	—
60‐69	13 (2.9)	3 (1.3)	0 (0.0)	—
Educational attainment, n (**%**)				.51
High school	65 (13.9)	40 (17.9)	7 (25.9)	—
Associate degree (2‐3-year college)	71 (16.0)	42 (18.8)	5 (18.5)	—
Bachelor’s degree (4-year university or higher)	269 (60.4)	126 (58.5)	14 (51.9)	—
Master’s degree	36 (8.1)	13 (5.8)	1 (3.7)	—
Doctoral degree	7 (1.6)	2 (0.9)	0 (0.0)	—
Marital status, n (**%**)				.11
Single	209 (47.0)	128 (57.4)	17 (63.0)	—
Cohabiting/common-law marriage	12 (2.7)	3 (1.3)	1 (3.7)	—
Married	219 (49.2)	88 (39.5)	9 (33.3)	—
Previously married (separated/divorced/widowed)	5 (1.1)	4 (1.8)	0 (0.0)	—
Current living arrangement, n (**%**)				.30
Living alone (1-person household)	66 (14.8)	36 (16.1)	7 (25.9)	—
Living with others (2 or more household members)	379 (85.2)	187 (83.9)	20 (74.1)	—
Subjective socioeconomic status, n (**%**)				.03
Lowest	10 (2.2)^c^	9 (4.0)^c^	2 (7.4)^c^	—
Low	98 (22.0)^c^	67 (30.0)^c^^,^ ^d^	12 (44.4)^d^	—
Middle	302 (67.9)^c^	135 (60.5)^c^	13 (48.1)^c^	—
High	33 (7.4)^c^	12 (5.4)^c^	0 (0.0)^c^	—
Highest	2 (0.4)^c^	0 (0.0)^c^	0 (0.0)^c^	—
Personality trait, mean (SD)				<.001
Openness to experience	3.16 (0.84)^c^	3.21 (0.88)^c^	2.90 (0.95)^c^	.20
Conscientiousness	3.90 (0.62)^c^	3.64 (0.68)^d^	3.39 (0.93)^e^	<.001
Neuroticism	2.61 (0.79)^c^	3.47 (0.84)^d^	3.95 (0.80)^e^	<.001
Extraversion	2.77 (0.82)^c^	2.67 (0.89)^c^	2.25 (0.78)^d^	.004
Agreeableness	3.49 (0.63)^c^	3.44 (0.65)^c^^, d^	3.17 (0.71)^d^	.04
Digital sensitivity, mean (SD)	90.13 (14.37)	90.49 (13.46)	86.74 (12.97)	.42
Smartphone operating system, n (**%**)				.31
iOS	151 (33.9)	80 (35.9)	13 (48.1)	—
Android	294 (66.1)	143 (64.1)	14 (51.9)	—

a Cluster 1, no or low risk; cluster 2, moderate risk; and cluster 3, high risk.

b Not available.

c Scores with the same superscript are not significantly different from each other but are significantly different from those with different superscripts.

d Scores with the same superscript are not significantly different from each other but are significantly different from those with different superscripts.

e Scores with the same superscript are not significantly different from each other but are significantly different from those with different superscripts.

According to the chi-square analyses for categorical variables, there were no significant differences among the 3 clusters in sex (*χ*²_2_=3.27, Cramér *V*=0.07; *P*=.20), age group (*χ*²_8_=8.42, Cramér *V*=0.08; *P*=.39), educational attainment (*χ*²_8_=7.21, Cramér *V*=0.07; *P*=.51), marital status (*χ*²_6_=10.29, Cramér *V*=0.09; *P*=.11), current living arrangement (*χ*²_2_=2.42, Cramér *V*=0.06; *P*=.30), and smartphone operating system (*χ*²_2_=2.34, Cramér *V*=0.06; *P*=.31). For continuous variables, separate 1-way ANOVAs revealed no significant group differences across the clusters in age (*F*_2, 692_=2.46, partial *η*²=0.007; *P*=.09) and digital sensitivity (*F*_2, 692_=0.86, partial *η*²=0.002; *P*=.42).

Among all variables examined, only SES and personality traits showed significant differences across the 3 clusters. For subjective SES, a significant group difference was observed (*χ*²_8_=17.41, Cramér *V*=0.11; *P*=.03). To compare column proportions, pairwise *z*-tests were conducted, and *P* values were adjusted using the Bonferroni method (*P*<.05). These comparisons revealed that the proportion of participants reporting a low SES level was significantly higher in cluster 3 (12/695, 44.4%) than in cluster 1 (98/695, 22.0%). In terms of personality traits, a 1-way MANOVA revealed a significant multivariate effect of group on the combined dependent variables (Pillai trace=0.26, *F*_10, 1378_=18.85, partial *η*²=0.13; *P*<.001). Among the Big Five personality dimensions, 1-way ANOVAs revealed significant group differences in conscientiousness (*F*_2, 692_=17.09, partial *η*²=0.05; *P*<.001), neuroticism (*F*_2, 692_=105.37, partial *η*²=0.23; *P*<.001), extraversion (*F*_2, 692_=5.61, partial *η*²=0.02; *P*=.004), and agreeableness (*F*_2, 692_=3.37, partial *η*²=0.01; *P*=.04), whereas no significant difference was found in openness to experience only (*F*_2, 692_=1.63, partial *η*²=0.005; *P*=.20).

As shown in Table 3, post hoc comparisons with Bonferroni correction indicated that both conscientiousness and neuroticism significantly differed across all 3 clusters (*P*<.001). Specifically, individuals in higher-risk clusters demonstrated lower levels of conscientiousness and higher levels of neuroticism than those in lower-risk clusters. Extraversion was significantly lower in cluster 3 than in both cluster 1 (*P*<.001) and cluster 2 (*P*=.04). For agreeableness, individuals in cluster 1 exhibited significantly higher scores than those in cluster 3 (*P*=.04), whereas no significant differences were found between cluster 2 and either of the other clusters.

#### Psychiatric Characteristics Across Clusters

To investigate differences in psychiatric characteristics across the 3 clusters, 1-way ANOVAs were conducted for each variable. As shown in Table 4, all psychiatric variables demonstrated significant group differences. Significant effects of cluster membership were found for the severity of depression (*F*_2, 692_=463.22, partial *η*²=0.57; *P*<.001), state anxiety (*F*_2, 692_=232.61, partial *η*²=0.40; *P*<.001), trait anxiety (*F*_2, 692_=273.99, partial *η*²=0.44; *P*<.001), insomnia (*F*_2, 692_=137.15, partial *η*²=0.28; *P*<.001), and somatization (*F*_2, 692_=197.63, partial *η*²=0.36; *P*<.001). Effect size interpretation based on partial *η*² indicated large effects for all variables (≥0.14). Post hoc analyses with Bonferroni correction indicated that all pairwise comparisons were statistically significant (*P*<.001) for depression, state and trait anxiety, and somatization. For insomnia, the difference was significant between cluster 1 and cluster 2 (*P*<.001) and between cluster 1 and cluster 3 (*P*<.001) but not between cluster 2 and cluster 3 (*P*=.07), indicating a similar level of insomnia severity between the moderate- and high-risk groups.

**Table 4.** Psychiatric characteristics of the study sample^a^.

Characteristics	Total (N=695)	Cluster 1 (n=445)	Cluster 2 (n=223)	Cluster 3 (n=27)	*P* value
Depression (PHQ-9^b^), mean (SD)	4.93 (5.71)	1.92 (2.53)^c^	9.34 (5.20)^d^	18.00 (5.66)^e^	<.001
State anxiety (STAI-X-1^f^), mean (SD)	44.59 (12.64)	38.85 (9.48)^c^	53.43 (10.26)^d^	66.07 (10.83)^e^	<.001
Trait anxiety (STAI-X-2), mean (SD)	45.09 (12.15)	39.26 (8.49)^c^	54.21 (10.04)^d^	65.85 (10.48)^e^	<.001
Insomnia (ISI-K^g^), mean (SD)	9.51 (6.28)	7.02 (4.95)^c^	13.67 (5.93)^d^	16.11 (5.98)^d^	<.001
Somatization (PHQ-15^h^), mean (SD)	7.34 (5.30)	5.02 (3.78)^c^	10.96 (4.87)^d^	15.44 (5.54)^e^	<.001

a Cluster 1, no or low risk; cluster 2, moderate risk; and cluster 3, high risk.

b PHQ-9: Patient Health Questionnaire–9.

c Scores with the same superscript are not significantly different from each other but are significantly different from those with different superscripts.

d Scores with the same superscript are not significantly different from each other but are significantly different from those with different superscripts.

e Scores with the same superscript are not significantly different from each other but are significantly different from those with different superscripts.

f STAI: State-Trait Anxiety Inventory.

g ISI-K: Korean version of the Insomnia Severity Index.

h PHQ-15: Patient Health Questionnaire–15.

## Discussion

### Principal Findings

The main findings of this study are as follows. First, we confirmed the superior sensitivity of mobile EMA for monitoring daily depressive symptoms compared with traditional retrospective scales. Second, our analysis revealed distinct temporal changes in depressive symptoms and patterns of recall consistency between the FW and SW of observation. Third, we identified specific subgroups at heightened risk for depression and suicidal ideation and characterized their unique sociodemographic, psychological, and psychiatric profiles. These results provide a multidimensional understanding of symptom dynamics and underscore the clinical usefulness of high-frequency digital monitoring for effective risk stratification among community-dwelling Korean adults.

This study provides empirical evidence that traditional retrospective reports systematically underestimate symptomatic experiences compared with daily EMA, which captures the simple daily recording of symptom presence. We found that a substantial portion of daily-level symptomatic variance is lost as participants are required to mentally reconstruct their experiences and estimate the total number of symptomatic days over successive recall periods. This process of heuristic estimation introduces a “smoothing effect” that obscures the true density and temporal complexity of depressive symptoms, resulting in significant information loss. Consequently, our findings suggest that retrospective frequency estimates insufficiently capture the full burden of depression, as the nuanced symptomatic fluctuations captured by prospective monitoring are flattened during memory-based aggregation.

What is more, we identified a paradoxical divergence in symptomatic patterns from the FW to the SW of assessment. While retrospective assessments indicated a significant decrease in depression severity scores, the prospective probability of actual symptom reporting concurrently increased. If this decrease in scores were due to regression to the mean, symptom reporting should have also decreased. Instead, the observed increase supports a psychological shift in symptom sensitization rather than a mere statistical artifact. This discrepancy is closely linked to intraindividual dynamics, which account for the vast majority of symptomatic variability and remain largely unexplained by traditional retrospective recall methods. Ultimately, the interaction between assessment week and recall consistency provides robust evidence that recall bias is not a fixed or static error; rather, it is a dynamic process associated with changes in a participant’s internal standards and cognitive framework for symptom recall during sustained daily monitoring.

Last but not least, this study highlights the value of digital phenotyping by identifying 3 distinct clinical clusters with diverging symptomatic patterns. While both mean symptom severity and suicidal ideation frequency increased linearly across the 3 risk groups, within-person symptom variability (SD) exhibited a nonlinear pattern, particularly peaking in the moderate-risk group. This peak in symptom variability may reflect the underlying complexity of ambivalent self-schemas in moderate-risk individuals. Furthermore, these clusters were validated by psychiatric and personality profiles, with the high-risk group distinguished by the highest neuroticism, the lowest conscientiousness, and a higher prevalence of low subjective SES. Collectively, these findings underscore within-person symptom dynamics as a critical feature in characterizing heterogeneous depression phenotypes and their associated suicide risk, both of which are often masked by the traditional retrospective assessments.

### Comparison With Prior Work

In line with previous findings that EMA has greater sensitivity in detecting depressive symptoms and suicidal ideation than retrospective self-report scales [18-20], this study suggests that traditional retrospective reports are not merely imprecise but systematically distorted. Indeed, the substantial information loss inherent in traditional 2-week recall was evidenced by its limited explanatory power, accounting for only 35.5% of the variance in actual daily symptom presence (model 1). The significant negative slope observed (*Β*=−0.406; *P*<.001) further indicates a fundamental misalignment between memory-based reports and actual daily occurrences. Whereas the traditional K-CESD-R scores were significantly higher for the FW than for the SW, the actual probability of daily symptom presence was found to be significantly higher in the SW than in the FW (0.908 vs 0.853; model 2). This discrepancy is likely rooted in cognitive heuristics; specifically, this inverse relationship suggests that retrospective recall is not a simple summary of experiences but is heavily distorted by specific intense episodes from the past, reflecting the “peak bias” of the peak-end rule [44]. In this case, participants appeared to rely on intense MDEs from the more distant past (FW) to calibrate their overall 2-week symptom reports, thereby overriding the actual increase in symptom frequency observed in the more recent period (SW). Given that nearly 79% of symptomatic variability is linked to intraindividual fluctuations, mobile EMA serves as a cognitively robust alternative that captures these dynamic patterns which are otherwise lost to recall bias.

Beyond identifying these recall biases, the observed paradoxical divergence between the decrease in retrospective scores and the increase in the daily probability of symptom reporting highlights shifting patterns in how participants perceive and evaluate their affective states over the course of the study. Engagement with EMA is associated with a refinement of internal standards for symptom appraisal, which may be related to heightened emotional awareness and behavioral reflection [22,23]. These results align with the concept of assessment reactivity [24], indicating that the act of frequent monitoring can be reflected in reporting patterns by sensitizing participants to their subtle symptomatic fluctuations. Therefore, mobile EMA provides a more ecologically valid reflection of symptomatic trajectories than traditional static measures, capturing the daily engagement with their own symptoms. While such heightened awareness may facilitate self-regulated recognition, it remains critical to acknowledge that increased self-focus or reinforced negative symptom monitoring may exacerbate psychological distress in certain individuals [25,26]. Overall, these findings support the potential usefulness of mobile EMA as a fine-grained tool for capturing the complexity of symptom trajectories.

To address inconsistent findings on daily negative affect dynamics across levels of depression severity [9,12,45], we classified community-dwelling participants into 3 distinct subgroups with depressive symptom dynamics: (1) cluster 1 (no or low risk; 445/695, 64.0%): low symptom severity, low symptom variability, and minimal suicidal ideation; (2) cluster 2 (moderate risk; 223/695, 32.1%): moderate symptom severity, the highest symptom variability, and occasional suicidal ideation; and (3) cluster 3 (high risk; 27/695, 3.9%): the highest symptom severity, moderate symptom variability, and frequent suicidal ideation. As hypothesized, within-person symptom variability, among the 3 active EMA features, played a key role in distinguishing not only between cluster 2 and cluster 3 but also between cluster 1 and clusters 2 and 3, as validated by the significant differences in the PHQ-9 scores across the 3 clusters. In line with the self-schema model for depression [15,16], these findings suggest that the highest symptom variability observed in cluster 2 may be attributed to the underlying instability of ambivalent self-schema representations, characterized by the coexistence of both positive and negative self-referent information. Importantly, the finding that higher variability was not always associated with greater symptom severity further suggests the presence of heterogeneous depression profiles.

Although cluster 3 (27/695, 3.9%) represented a relatively small proportion of the total sample, this distribution aligns with the expected epidemiological prevalence of high-risk individuals with suicidal ideation in the South Korean general population. Recent national surveys report that the proportion of adults who experienced suicidal thoughts within the past year ranges from 2.1% (2023 National Survey on Suicide [46]) to 4.6% (2023 Korea National Health and Nutrition Examination Survey [47]). The high degree of consistency between our findings (27/695, 3.9%) and these national representative data strongly supports the ecological validity of this cluster analysis, suggesting that cluster 3 is not a statistical outlier but a clinically valid subgroup of the at-risk population in a real-world context. Moreover, while national statistics often estimate suicide risk based on a 1-year history of suicidal ideation without considering depression severity, this study evaluated the co-occurrence of both active suicidal thoughts and depressive symptoms within a brief 2-week window. Furthermore, consistent with the previous study [21], mobile EMA may reduce the concealment of suicidal thoughts typically encountered in retrospective assessments and identify highly specific, high-risk phenotypes, providing a more sensitive clinical snapshot of acute suicidal risk associated with depression.

As well as the PHQ-9 scores, the STAI-X-1, STAI-X-2, and Patient Health Questionnaire–15 scores significantly differed across the clusters, showing a consistent increase with higher-risk levels (cluster 1<2<3). Consistent with the previous findings that individuals with depressive symptoms are more vulnerable to insomnia than those with no depressive symptoms in a general population–based sample from the Republic of Korea [48], this study also observed an increase in the ISI-K scores in accordance with the cluster risk level. Furthermore, the lack of a significant difference in the ISI-K scores between clusters 2 and 3 aligns with prior evidence suggesting that the severity of depressive symptoms is not significantly influenced by the presence or severity of insomnia symptoms among those with depressive symptoms [48]. In this regard, the risk levels assigned to each cluster closely corresponded to their psychiatric characteristics, providing theoretical support for the association between symptom variability and the instability of depressive self-schema.

In addition to psychiatric profiles, the cluster classification was also associated with sociodemographic factors and personality traits known as risk factors for both depressive symptoms and suicidality. Consistent with the previous findings that low subjective SES predicted higher levels of depressive symptoms and suicidal ideation [49,50], cluster 3 (12/27, 44.4%) accounted for a significantly higher proportion of individuals with low subjective SES than cluster 1 (98/445, 22.0%), showing that their proportion increased with the cluster risk level. Interestingly, even in cluster 3 (the high-risk group), only a small proportion of participants reported the lowest level of subjective SES, while those with low (but not the lowest) SES were overrepresented. This pattern supports prior findings that relative deprivation, rather than absolute poverty, is more closely associated with depression and suicidal risk [51,52]. These findings also align with the social rank theory of depression [53], which posits that lower perceived social status increases vulnerability to depression and suicidality.

Among the Big Five personality traits, neuroticism, conscientiousness, extraversion, and agreeableness significantly differed across the 3 clusters. Individuals in the high-risk cluster exhibited the highest neuroticism and the lowest levels of conscientiousness, extraversion, and agreeableness. Consistent with prior evidence [54-56], neuroticism and conscientiousness were the personality traits that most clearly distinguished the 3 clusters. Particularly, cluster 3 exhibited the highest levels of neuroticism and the lowest levels of conscientiousness. These findings suggest that those in cluster 3 are more prone to emotional instability including heightened sensitivity to stress and frequent negative emotions, such as anxiety and anger, and experience greater difficulties with self-regulation in impulse control, organization, and goal-directed behavior. Although extraversion and agreeableness also differed across some of the clusters, both traits were not as clearly discriminative of depression risk as neuroticism and conscientiousness [57,58]. Extraversion was significantly lower in cluster 3 than in clusters 1 and 2, whereas agreeableness was significantly lower than in cluster 1 but did not differ from cluster 2. The observed reductions in extraversion and agreeableness among individuals in cluster 3 may reflect increased social withdrawal and interpersonal difficulties, which are commonly associated with depressive symptomatology [3]. Taken together, the current findings highlight the importance of neuroticism and conscientiousness in differentiating depression risk profiles, while emphasizing the added value of interpersonal traits such as extraversion and agreeableness in identifying individuals at high risk for social and emotional dysfunctions associated with increased vulnerability to depression and suicidality.

### Limitations and Future Directions

This study provides quantitative evidence of systematic recall bias and the clinical usefulness of digital phenotyping. Despite its strengths, the following limitations should be considered when interpreting the results of this study. Most of all, specific methodological limitations regarding the temporal scope of the study, including the frequency of daily assessments and the overall duration of observation, warrant consideration. First, although mobile EMA offers high ecological validity, a 24-hour recall format used in this study may still be subject to reporting biases (eg, underreporting or momentary inattention) and have missed more granular, in-the-moment fluctuations that occur throughout the day. Particularly, the present EMA framework may still have been insufficient to capture the diurnal variability and transient surges of suicidal thoughts, which are often short-lived yet clinically significant. Second, it is true that the identification of the relatively small, high-risk group (27/695, 3.9%) underscores the superior sensitivity of mobile EMA in capturing the dynamic nature of suicidal risk that might otherwise be underreported in conventional retrospective assessments. However, the brief 2-week observation window represents only a snapshot of the dynamic nature of suicidal ideation in depressed individuals. Since suicidal ideation can be reactive to transient environmental stressors or life events, the long-term stability of this high-risk phenotype remains to be established, suggesting that cluster assignments might vary over a more extended longitudinal period.

Beyond these temporal considerations, additional limitations involving our analytical procedures, study design, and potential measurement effects also require careful interpretation. Third, the exclusion of 203 participants, who failed to complete the midpoint K-CESD-R survey (day 7), may have introduced selection bias. Although this criterion was applied to maintain the continuity of depressive symptoms, which is a key diagnostic criterion for MDD, the primary reason for this exclusion was adherence to the IRB-approved study protocol, which mandated the disposal of data from participants who withdrew or were lost to follow-up. Accordingly, we were not able to perform sensitivity analyses to compare the excluded and retained groups, which remains a limitation in confirming the robustness of our results against potential attrition bias. Fourth, although we observed significant decreases in week-to-week symptom severity scores across 2 different assessment methods, the observational design precludes causal inferences regarding the effects of EMA-based self-monitoring on symptom improvement. Finally, the repeated use of the same depression scale (K-CESD-R) in both online and mobile formats may have had a carryover effect, potentially improving participants’ symptom recall over time and reducing recall bias in the traditional online version.

Several research directions are suggested to overcome these methodological limitations. To improve the limited temporal resolution of a 24-hour recall format, follow-up protocols could incorporate higher sampling frequencies, such as multiple assessments per day, to more precisely capture transient surges of suicidal ideation. Expanding the observation window through longitudinal study designs that span more than 2 weeks will also be essential to establish the long-term stability of the identified phenotypes. Furthermore, implementing more flexible data retention policies in coordination with IRB would allow for the inclusion of partial data in sensitivity analyses, which in turn would minimize selection bias. The integration of real-time passive sensing (eg, step count, sleep time, GPS, screen time, call or app logs, etc) with active EMA could further enhance predictive precision while alleviating participant burden. To establish the causal efficacy of EMA-based self-monitoring, future studies should also use randomized controlled trials comparing EMA with appropriate control conditions. Finally, future research should replicate this methodological approach using other validated depression scales to determine whether similar patterns of recall-related underreporting are observed, thereby addressing potential carryover effects from repeated measurements. Such efforts to establish convergent evidence would significantly improve the reliability and generalizability of findings regarding recall bias.

### Conclusions

This study demonstrates that mobile EMA provides more sensitive and ecologically valid monitoring of depressive symptoms than traditional retrospective measures. Conventional assessments are subject to both systematic underreporting (recall bias) and limited sensitivity to daily symptom variability. Further week-to-week analysis revealed a significant reactivity effect, with daily symptom reporting increasing over the 2-week period. This finding suggests that EMA acts as a mechanism to enhance self-awareness. Finally, active EMA features, including symptom severity, symptom variability, and frequency of suicidal ideation, effectively identified integrated depression and suicide risk subgroups and their distinct sociodemographic, psychological, and psychiatric profiles, highlighting the usefulness of digital phenotyping. Among these markers, symptom variability emerged as a key indicator for differentiating moderate-risk individuals, providing more detailed information than traditional measures. These findings pave the way for more personalized and timely mental health assessments and interventions in clinical practice.

## Supplementary material

10.2196/80332 Checklist 1 STROBE (Strengthening the Reporting of Observational Studies in Epidemiology) checklist for observational studies in epidemiology (cross-sectional).

## Abbreviations

AICC: Akaike Information Criterion Corrected
BIC: Bayesian Information Criterion
CESD-R: Center for Epidemiologic Studies Depression Scale-Revised
CONSORT: Consolidated Standards of Reporting Trials
*DSM-5*: *Diagnostic and Statistical Manual of Mental Disorders*, Fifth Edition
EMA: ecological momentary assessment
FW: first week
GLMM: Generalized Linear Mixed Model
ICC: intraclass correlation coefficient
IRB: institutional review board
ISI: Insomnia Severity Index
ISI-K: Korean version of the Insomnia Severity Index
K-CESD-R: Korean Version of the Center for Epidemiologic Studies Depression Scale–Revised
MDD: major depressive disorder
MDE: major depressive episode
PHQ-9: Patient Health Questionnaire–9
SES: subjective socioeconomic status
STAI: Spielberger State-Trait Anxiety Inventory
STROBE: Strengthening the Reporting of Observational Studies in Epidemiology
SW: second week
